# Largely Intact But Less Reliable and Distributed Neural Representations of Subjective Value in Human Opioid Addiction

**DOI:** 10.1523/JNEUROSCI.0679-25.2025

**Published:** 2025-09-11

**Authors:** Francesca M. LoFaro, Maëlle C. M. Gueguen, Ananya Kapoor, Emmanuel E. Alvarez, Darla Bonagura, Anna B. Konova

**Affiliations:** ^1^Department of Psychiatry, University Behavioral Health Care, & the Brain Health Institute, Rutgers University, Piscataway, New Jersey 08854; ^2^Laureate Institute for Brain Research, Tulsa, Oklahoma 74136; ^3^Oxley College of Health and Natural Sciences, University of Tulsa, Tulsa, Oklahoma 74136; ^4^Department of Biology, Institute of Ecology and Evolution, University of Oregon, Eugene, Oregon 97403; ^5^Department of Psychology, University of Tennessee, Knoxville, Tennessee 37996

**Keywords:** addiction, decision-making, fMRI, multivariate decoding, subjective value, uncertainty

## Abstract

Addiction, particularly opioid use disorder (OUD), is often characterized by heightened propensity for risk-taking. While tolerance for risk and uncertainty varies across individuals, the elevated risk-taking in people with OUD is assumed to stem from altered cognitive decision-making processes beyond differences due to idiosyncratic yet lawful tolerances. Specifically, the prevailing assumption is that people with addiction exhibit impairments in the internal representation and integration of information that should guide decisions and judgments about what is valuable. Using model-based functional magnetic resonance imaging, we examined how the choice behavior of treatment-engaged male and female participants with chronic OUD aligns with the neural encoding of their inferred subjective value (SV) of uncertain (risky and ambiguous) rewards and the evidence for impairment in this neural process. Using both univariate and multivariate analyses, we found that canonical value regions [ventromedial prefrontal cortex (vmPFC), striatum, and posterior cingulate cortex] track the SV of uncertain choice options in both participants with OUD and comparison controls, irrespective of their tolerance for uncertainty. This speaks against a fundamentally impaired subjective valuation process in OUD. However, value representations were less reliably decodable in people with OUD in some value regions (vmPFC) and throughout the brain, especially within the limbic and salience/ventral-attention networks. Thus, while people with OUD engage a neurocomputationally similar process during risky decision-making to controls, they may differ in the fidelity and distribution of SV signals across brain networks.

## Significance Statement

A common assumption in addiction neuroscience is that people with substance use disorders have impaired encoding or computation of the value of their options and consequently might engage in risky behaviors over less risky alternatives. Empirical support for this viewpoint however remains lacking, potentially hindering the translation of research into improved understanding and treatment of addiction. This study shows the valuation of risky decisions is largely neurally intact, though less reliable and distributed throughout the brain, in people with opioid use disorder. Thus, rather than impaired valuation, addiction may be associated with a less robust, and spatially more restricted, representation of value. These findings highlight network-level mechanisms that shape decision-making and suggest new targets for future addiction research and treatment.

## Introduction

Substance use disorders are clinically defined by criteria such as using more of a substance than intended, recurrent use in risky and hazardous situations, and use despite knowledge of adverse health, social, and interpersonal impacts (APA, 2013). Among the spectrum of substance use disorders, opioid use disorder (OUD) stands out for its severe health and societal consequences observed during the ongoing opioid crisis. These aspects of addictive behavior, and OUD in particular, are generally interpreted as failures to appropriately incorporate information about risks in assessing the value of candidate actions, leading to maladaptive decisions and risk-taking (Helie et al., 2017; Verdejo-Garcia et al., 2019; Bickel et al., 2020; Kluwe-Schiavon et al., 2020). Indeed, numerous studies have found that people with addiction, including OUD, demonstrate an increased propensity for risk-taking across a range of experimental paradigms (Brand et al., 2008; Lawrence et al., 2009; Gowin et al., 2013; Chen et al., 2020; Konova et al., 2023) and that this behavior may render individuals more vulnerable to relapse (Passetti et al., 2008; Verdejo-Garcia et al., 2018; Konova et al., 2020) and other adverse health outcomes (McCoy et al., 2004; Armoon et al., 2023). Yet, these differences in risk-taking propensity have not, so far, been directly linked to an underlying alteration in the neural computation or encoding of value during risky choice.

Of course, more than just people with addiction take risks. Almost every decision that we make involves a degree of uncertainty. Economists and decision neuroscientists have generally considered two contexts for uncertainty: “known-risk,” when the probabilities associated with uncertain outcomes are known (e.g., a 50% chance at winning $100), and “ambiguity,” when these probabilities are unknown or partially unknown and cannot be estimated (e.g., a 30–70% chance at winning $100). Extensive evidence suggests that people vary significantly in their tolerance (also referred to as preferences) for both known-risk and ambiguity and that these tendencies tend to only weakly correlate (Levy et al., 2010; Tymula et al., 2012; Pushkarskaya et al., 2015; Ruderman et al., 2016; Konova et al., 2020; Raio et al., 2022). Together, these preferences are thought to contribute to an individual's global risk-taking propensity.

Decision neuroscience research has identified a domain-general neural valuation circuit involved in the computation of value across contexts, reward modalities, and stages of the decision-making process, consisting of the ventromedial prefrontal cortex (vmPFC), striatum, and posterior cingulate cortex (PCC; Levy and Glimcher, 2012; Bartra et al., 2013; Clithero and Rangel, 2014; Lopez-Persem et al., 2020), which encodes choice-relevant subjective value (SV) signals that are calibrated by individual tolerances for uncertainty (Levy et al., 2010; Konova et al., 2023), similar to other idiosyncratic tendencies (e.g., tolerance for delay; Kable and Glimcher, 2007). Thus, if elevated risk-taking in addiction stems from altered cognitive decision-making processes beyond differences due to idiosyncratic but nonetheless lawful tolerances, it is possible that this canonical value circuit is not adequately integrating information to compute value, is not reliably encoding the computed value, or both, thus resulting in maladaptive (and often excessively risky) decisions. If this is the case, using an economic risky decision-making task and SV model combined with functional magnetic resonance imaging (fMRI), we should observe misalignment between OUD participants’ behavioral risk-taking propensities and engagement of neural valuation processes.

Importantly, value is an influential modulatory factor for a broad range of cognitive and sensory processes, in addition to risky decision-making. Thus, while we expect to find the above evidence for disrupted subjective valuation under uncertainty in canonical value regions, building on prior findings of broadly distributed decision-related activity (Vickery et al., 2011; Hunt and Hayden, 2017; Overton et al., 2025), we also assessed for more ubiquitous value signals throughout the brain, including limbic, sensory, and motor cortices. Therefore, in this study, we tested how both canonical value regions and regions spanning multiple large-scale brain networks compute/track SV to guide decision-making in individuals with chronic OUD and comparison controls.

## Materials and Methods

### Participants

Participants were individuals receiving medications for OUD (primarily buprenorphine) as treatment for opioid addiction at two university-affiliated intensive outpatient programs and comparison controls recruited from the same geographic area through flyers, online ads, and word of mouth. We focused on this population because, despite the well-established benefits of medications for OUD, opioid reuse and other “risky” behaviors remain common among people receiving these gold-standard treatments. This persistent vulnerability suggests these behaviors are not being fully addressed by the treatment, as supported by meta-analytic data that show that differences in risky decision-making between individuals with OUD and healthy controls are robust, even in samples where the majority (>70%) are receiving medications for OUD (Biernacki et al., 2016).

Sixty-five participants were enrolled and completed the study procedures. All participants provided written informed consent in accordance with the Rutgers University Institutional Review Board. Inclusion criteria for both groups were (1) 18 years or older and (2) the ability to read and understand English. OUD participants additionally needed to have (3) a diagnosis of OUD, as determined by DSM criteria and ascertained through patient charts, encompassing heroin and/or painkiller use; (4) at least a 12 month history of opioid use; (5) and current receipt of medications for OUD treatment on an outpatient basis. Exclusion criteria for both groups were (1) active psychosis or mania or lifetime history of schizophrenia; (2) history of intellectual disability or developmental or neurological disorder, seizures or epilepsy, or loss of consciousness lasting >30 min; (3) severe medical conditions requiring hospitalization or that, in the opinion of the study staff, could compromise study participation; (4) MRI contraindications (claustrophobia, nonremovable piercings, metal in the body, etc. and pregnancy); and (5) failure to understand or comply with study procedures. Additional control participant-specific exclusion criteria were (6) history of substance abuse (except for nicotine and alcohol use restricted to college or military service); (7) a positive urine drug screen for any psychoactive substances or their metabolites on any study day; and (8) the use of central nervous system medications within the past 6 weeks.

Inclusion and exclusion criteria for enrollment were ascertained during a comprehensive screening, which included the Addiction Severity Index (McLellan et al., 1992), Beck Depression Inventory (Beck et al., 1996), State–Trait Anxiety Inventory (STAI; Spielberger and Gorsuch, 1983), and a medical history checklist, and by consulting clinic records for OUD participants. At the MRI session (completed on a separate day), for all participants, we additionally assessed state anxiety with the STAI-S and performed a urine drug screen for common substances and their metabolites including opioids, cocaine, cannabis, and benzodiazepines, among others. For OUD participants, we also assessed opioid-specific signs and symptoms of withdrawal with the Subjective Opiate Withdrawal Scale (SOWS; Handelsman et al., 1987); current craving, urge for, and ability to resist using heroin with the Heroin Craving Questionnaire (HCQ; Tiffany et al., 1993); and self-reported opioid and other substance use (including medications for OUD adherence) in the 7 d prior to the session with the Timeline Follow-Back (Sobell and Sobell, 1992).

From *N* = 65 participants who completed the study procedures, five were excluded from all analyses. Three participants (*n* = 2 OUD; *n* = 1 control) were excluded for excessively random/noisy task behavior, demonstrating they did not understand or sufficiently attend to the task, and two (*n* = 2 controls) were excluded for choosing the safe option on >95% of trials. These behavioral patterns resulted in extreme preferences that were not adequately captured by the model and influential outliers during fMRI analysis. Therefore, *n* = 33 OUD participants (seven females; mean [SE] age = 45.12 [2.165] years) and *n* = 27 comparison controls (11 females; mean [SE] age = 44.96 [2.870] years) were retained for analysis. Groups were matched on age, sex, race, and ethnicity, but they differed in education, income, numeracy, and nonverbal IQ (although not working memory) and depression and anxiety symptoms (Table 1).

**Table 1.** Sociodemographic and clinical characteristics of the study sample

	OUD		Controls		Test	*p*
	*N* = 33		*N* = 27			
Sociodemographic and cognitive						
Age (years)	*M* = 45.12	SE = 2.17	*M* = 44.96	SE = 2.87	*t* = 0.04	0.96
Sex:						
Male	26		16		*χ*^2^ = 2.70	0.10
Female	7		11			
Race:						
Asian	0		1		*χ*^2^ = 3.82	0.28
Black or African American	17		8			
White or Caucasian	13		15			
Other or More Than One Race	3		3			
Ethnicity:						
Hispanic	6		7		*χ*^2^ = 0.52	0.47
Non-Hispanic	27		20			
Education:						
Primary complete	4		1		*χ*^2^ = 32.23	<0.001
High school complete/GED	22		1			
Some college or college complete	7		19			
Some graduate or graduate complete	0		6			
Monthly income	$0–$9,000 (Median=$464)		$0–$10,000 (Median=$2,000)		*z* = −2.79	<0.01
Nonverbal IQ: K-BIT^a^	*M* = 91.70	SE = 1.92	*M* = 105.77	SE = 3.13	*t* = −3.95	<0.001
Numeracy: module of the HHS (0–6)^b^	*M* = 3.03	SE = 0.27	*M* = 4.26	SE = 0.27	*t* = −3.21	<0.01
Working memory: Digit Span reverse^c^	*M* = 6.17	SE = 0.43	*M* = 6.23	SE = 0.34	*t* = −0.11	0.91
Psychiatric and substance use						
Depression: BDI-II (0–63)^d^	*M* = 18.19	SE = 2.05	*M* = 5.48	SE = 1.18	*t* = 5.03	<0.001
Trait Anxiety: STAI-T (20–80)^e^	*M* = 44.42	SE = 2.12	*M* = 36.15	SE = 2.22	*t* = 2.65	<0.05
Current Anxiety: STAI-S (20–80)^f^	*M* = 31.39	SE = 1.96	*M* = 28.38	SE = 1.74	*t* = 1.11	0.27
Nicotine Use: FTND (0–10)	*n* = 27		*n* = 1		*t* = 1.49	0.15
	*M* = 7.04	SE = 0.23	*M* = 5	*–*		
Alcohol use (lifetime years)	*n* = 32		*n* = 22		*t* = −1.34	0.19
	*M* = 15.32	SE = 2.31	*M* = 20.96	SE = 3.72		
Cocaine use (lifetime years)	*n* = 29		–		–	–
	*M* = 10.52	SE = 1.63	–	–		
Opioid use (lifetime years)	*M* = 16.82	SE = 2.17	–	–	–	–
Intravenous use (%)	33.33		–		–	–
History of overdose (%)	40.63		–		–	–
Hepatitis C positive status (%)	18.18		–		–	–
HIV positive status (%)	3.03		–		–	–
Duration of current treatment (months)	0.37–36 (Median = 8 months)		–		–	–
Treatment medication:	Dose (mg)		–		–	–
Suboxone/buprenorphine (*n* = 30)^g^	*M* = 18.69	SE = 1.43				
Methadone (*n* = 2)	*M* = 93.00	SE = 7.00				
Vivitrol/naltrexone (*n* = 1)	50.00	–				
Duration of opioid abstinence (days)^h^	0–2,694 (Median = 3 d)		–		–	–
Past 7 d opioid use (% yes)	57.58		–		–	–
Current Craving: HCQ-NOW (1–7)^h,i^	*M* = 2.58	SE = 0.35	–	–	–	–
Current Withdrawal: SOWS (0–64)^h^	*M* = 4.63	SE = 1.06	–	–	–	–

a Kaufman Brief Intelligence Test (K-BIT). Normative data suggest scaled scores between 85 and 115 constitute the average nonverbal IQ of the population. K-BIT data missing for *n* = 1 control.

b Numeracy module of the Health and Human Services (HHS) survey.

c Working memory test - Digit Span reverse (DSMT). DSMT-reverse score missing for *n* = 3 OUD participants and *n* = 1 control.

d Beck Depression Inventory (BDI-II). Depression severity cutoffs for the BDI-II are as follows: 0–13 minimal, 14–19 mild, 20–28 moderate, and 29–63 severe. BDI-II data missing for *n* = 2 OUD participants.

e State–Trait Anxiety Inventory-Trait and State (STAI-T and STAI-S). Anxiety severity cutoffs for the STAI-T are as follows: 20–37 no or low anxiety, 38–44 moderate anxiety, and 45–80 high anxiety. STAI-T data are missing for *n* = 2 OUD participants.

f STAI-S data is missing for *n* = 1 control.

g Buprenorphine medication dose is missing for *n* = 1 OUD participant.

h Abstinence duration and current craving and withdrawal data are missing for *n* = 1 OUD participant.

i Current craving is defined as the average of the (reverse-scored) “craving now,” “urge now,” and “resist now” items from HCQ-NOW.

### Experimental design

#### Risky decision-making task and computational modeling of individual preferences

**Figure 1.**
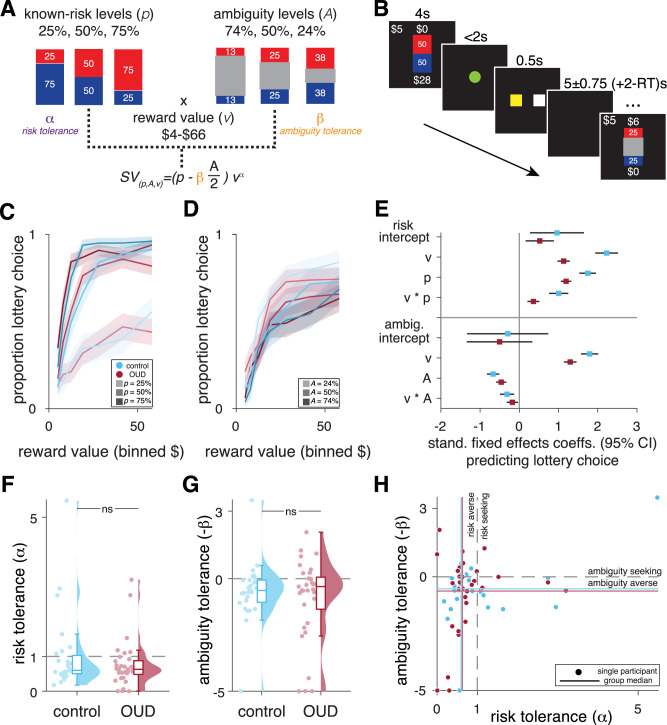
Risky decision-making task and behavior. ***A***, Participants chose between a sure $5 and playing a lottery for another amount (*v*) that could be received with either one of the three known probabilities (*p*) (25, 50, or 75; known-risk context) or one of three partially unknown probabilities determined by an ambiguity level (*A*) (74, 50, or 24%; ambiguity context). A modified utility model was fit to participants’ trial-by-trail choices to estimate individual risk and ambiguity tolerance parameters that inform SV. ***B***, Example trial sequence. Following display of the lottery and safe options (4 s), participants were instructed to make a choice upon seeing a central cue turn green (2 s). A yellow square was then shown, confirming the chosen side (0.5 s). ***C***, ***D***, Mean (±SE) proportion lottery choice in comparison controls and participants with OUD, by reward value and uncertainty level, for (***C***) known-risk and (***D***) ambiguity trials. ***E***, Standardized coefficients (±95% CI) from generalized mixed-effect models predicting lottery choice based on lottery characteristics for (top) known-risk and (bottom) ambiguity trials. ***F***, ***G***, Model-based risk and ambiguity tolerance parameters by group, with ***H*** showing the two parameters were largely uncorrelated. Dots are individual participant data and sky plots represent group density functions. Boxplots represent median and interquartile range. Dashed gray lines in ***F–H*** represent risk- and ambiguity-neutral choosers. ns, not significant.

During fMRI, participants completed a risky decision-making task, adapted from prior studies (Levy et al., 2010; Tymula et al., 2012; Konova et al., 2020), and designed to capture a wide range of uncertainty preferences, including relatively extreme uncertainty-seeking and uncertainty-averse behavior. On each trial, participants chose between a certain (“safe”) $5 and playing a lottery. Each lottery had two possible outcomes, $0 or *v*, where *v* ranged from $4 to $66 (21 unique amounts: $4, $5, $6, $7, $8, $9, $10, $12, $14, $16, $18, $20, $23, $26, $30, $34, $39, $44, $50, $57, and $66). Half of the trials involved known-risk, with *v* associated with one of three explicitly stated probabilities (*p*: 25, 50, or 75%). For the other half involving ambiguity, the reward probability (which was always 50%) was partially occluded and therefore rendered ambiguous. We used three levels of ambiguity *A*: low (24% occlusion), medium (50% occlusion), and high (74% occlusion). Including two types of uncertainty in this task allows us to more completely capture an individual's global propensity for risk-taking by accounting for separable uncertainty contexts. Each amount *v* appeared with each uncertainty level (*p*, *A*) once in a random order over four blocks/runs of 30 trials, for 120 unique trials (Fig. 1*A*), except for *v* = $4 which was only shown at the beginning of each run with *p* set to 50% and no ambiguity. This resulted in a total of 124 trials. Each of the four task runs were 6 min and 4 s (total task duration of 25 min).

On each trial, participants were shown a display of the lottery option and safe option for 4 s before being presented with a central green circle during which time they could choose one of the options. Participants then had 2 s to make a choice. The choice made was confirmed with a 0.5 s display of a yellow-colored square matching the side of the chosen option. No outcomes were shown. A 5 ± 0.75 s + (2 s-RT) intertrial interval fixation separated the trials (Fig. 1*B*).

To incentivize participants to make decisions according to their true preferences, they were instructed that one trial from the task would be randomly selected and played out for a bonus ($0–$66), to be added to a baseline compensation of $30. The task was explained as each trial being a choice between a certain $5 bonus or “playing the lottery” whereby each lottery corresponded to a physical bag containing 100 red and blue chips with one color representing $0 and the other the value of the lottery (*v*). The bags were always nearby for participants to inspect after the scan if they so wished. For known-risk lottery bags, the exact number of red and blue chips in each bag was specified. For ambiguous lottery bags, only partial information was provided on the number of red and blue chips with the portion occluded corresponding to the ambiguity level (to eliminate bias, as noted above, the number of red and blue chips was made equal in these bags). Participants received extensive instructions on the task including a task comprehension quiz and 18 practice trials (both repeated as needed). After the scan, one trial was randomly selected, and the choice made on that trial determined the bonus ($5, $0, or the reward value of the lottery) with the participant picking one chip out of the corresponding bag if they chose to play the lottery to determine the bonus. No bonus was received for missed trials.

As in prior work (Levy et al., 2010; Tymula et al., 2012; Pushkarskaya et al., 2015; Ruderman et al., 2016; Konova et al., 2020), we modeled participants’ choice data with a modified power utility model that separately parameterized known-risk and ambiguity tolerance. Here, the SV of each option (safe or lottery) on each trial is given by as follows:

$$\mathrm{SV}=[p-\beta \left(\frac{A}{2}\right)]v^{\alpha },\;(1)$$

where *v* is the reward value ($5 or the lottery amount), *p* is the reward probability, *A* is the fraction of the lottery that is occluded on ambiguity trials, *α* is a participant-specific known–risk tolerance parameter, and *β* is a participant-specific ambiguity tolerance parameter. Risk neutrality is indicated by *α *= 1, while *α *< 1 indicates risk aversion and *α *> 1 indicates risk-seeking. Ambiguity neutrality is indicated by *β *= 0, while *β *> 0 indicates ambiguity aversion and *β *< 0 indicates ambiguity-seeking. Given the approximate interpretable parameter range for our choice set and to account for potential estimation instability and decision noise, we estimated *α* and *β* using a constrained maximum-likelihood estimation in MATLAB version R2024b (*fmincon* bounds, 0 ≤ *α* ≤ 10 and −5 ≤ *β* ≤ 5) and fitting a probabilistic choice function to the trial-by-trial data as follows:

$$Pr_{\left(\mathrm{lottery}\right)}=\;\frac{1}{1+\;e^{\;\gamma (SV_{\left(\mathrm{lottery}\right)}-SV_{\left(\mathrm{safe}\right)})}},\;(2)$$

where Pr is the probability the lottery is chosen; SV_(lottery)_ and SV_(safe)_ are the SVs (from Eq. 1) of the lottery and safe options, respectively; and *γ* is a third participant-specific parameter capturing choice stochasticity/noise (unbounded). For ease of interpretation, we report risk tolerance as *α* and ambiguity tolerance as −*β*, so that higher values indicate higher tolerance for both parameters.

#### MRI data acquisition and preprocessing

Scanning was performed on a Siemens TRIO using a 32-channel head coil and multiband imaging. Structural scans, used in data preprocessing, consisted of a T1w MPRAGE image with 0.8 mm isotropic voxels, 256 FOV, 192 slices, 8° flip angle, 2,400 ms TR, 2.31 ms TE, and in-plane acceleration GRAPPA factor of 2 and a T2w SPC image with 0.8 mm isotropic voxels, 256 FOV, 208 slices, 3,200 ms TR, 566 ms TE, and in-plane acceleration GRAPPA factor of 2. For task-based scans, blood oxygenation level-dependent (BOLD) sensitive T2*-weighted images were acquired with 2.4 mm isotropic voxels, 230 FOV, 56 slices, 52° flip angle, 1,000 ms TR, 34.8 ms TE, a multiband factor of 7, and no in-plane acceleration (i.e., GRAPPA) and by tilting the slice acquisition 20° from each participant's AC–PC line to maximize coverage of the vmPFC. In addition, two brief (four volumes) spin echo images were acquired with geometry matching the functional data, one in the same phase encoding direction (AP) and the other in the opposite direction (PA), used in data preprocessing to correct for magnetic field distortions in the functional data.

Raw neuroimaging data were converted to Brain Imaging Data Structure format using heudiconv (https://github.com/nipy/heudiconv), preprocessed, and quality checked with fMRIPrep 20.2.7 (Esteban et al., 2019), which is based on Nipype 1.7.0 v (Gorgolewski et al., 2011; Esteban et al., 2019). Structural images were intensity normalized and skull-stripped and spatially normalized and registered to the MNI152NLin2009cAsym template. Functional images were distortion-, motion-, and slice-time corrected and coregistered to participants’ structural images using standardized workflows in fMRIPrep. Functional data were resampled to 2 mm^3^ and spatially smoothed with a 6 mm^3^ full-width at half-maximum Gaussian kernel, except data used for multivariate analyses which remained unsmoothed.

All data were visually inspected prior to analysis. We considered a participant's task run to have potentially problematic motion if they had >10% of volumes with 1.2 mm framewise displacement or higher. While two participants each had a single run exceeding this threshold, the removal of these runs did not change any of the results. Therefore, their complete dataset was retained in the fMRI analyses. The percentage of volumes per run with a framewise displacement exceeding the above threshold was 1.18 ± 2.56% in controls and 0.88 ± 1.84% in OUD participants (*t*_(58)_ = −0.634; *p* = 0.529). Mean framewise displacement across runs similarly did not differ between groups (*t*_(58)_ = −0.498; *p* = 0.621).

### Statistical analyses

Although OUD and control participants differed on several sociodemographic and cognitive factors and depressive and anxiety symptoms (Table 1), these variables did not significantly influence task behavior or neural responses, and therefore we did not include them as covariates in the analyses reported.

#### Behavior

To determine the marginal effects of the task characteristics on lottery choice, we used generalized linear mixed-effect models. These model-free analyses (one for known-risk trials and one for ambiguity trials) predicted participants’ trial-by-trial choices (selecting the lottery or safe option) from the probability level (*p*) or ambiguity level (*A*) associated with the lottery option, respectively, lottery value (*v*), and the interaction terms v*p or v*A and both by diagnosis, including random intercepts and slopes for main effects per participant. The probability level, ambiguity level, and reward value were *z*-scored to standardize the model output estimates. Models were fitted using Laplace approximation and a logit-link function in MATLAB version R2024b with *fitglme*. Missing data were not imputed and were censored in analyses.

Model-based risk and ambiguity tolerance, and choice stochasticity, were quantified by the best-fitting parameters for each participant (*α*, *β*, and *γ*) and compared between the diagnostic groups using two-tailed Wilcoxon rank-sum tests. Model fits (pseudo-*R*^2^) were similarly compared between groups with two-tailed two–sample *t* tests. Model-free behavior, or average proportion of lottery choices, was compared with corresponding model-based behavior using two-tailed Spearman correlations; this was similarly done to assess the relationship between participants’ known-risk tolerance (*α*) and their ambiguity tolerance (*β*).

#### Univariate BOLD fMRI analyses

To test hypotheses regarding neural computations of SV, the following first-level general linear models (GLMs) were estimated in SPM12 (https://www.fil.ion.ucl.ac.uk/spm). For all GLMs, we modeled task condition effects for the full 4 s decision period during which participants deliberated on their decision prior to registering a response (Fig. 1*B*). All task regressors were convolved with a canonical hemodynamic response function. In addition, each GLM included the following 13 nuisance regressors estimated during preprocessing with fMRIPrep: framewise displacement for each volume as a quantification of the estimated bulk-head-motion, six rigid-body motion–correction parameters (three translation and three rotation), and the first six noise components of the anatomical variant of CompCorr (aCompCorr). A standard high-pass filter cutoff was set at 1/128 s.

**Figure 2.**
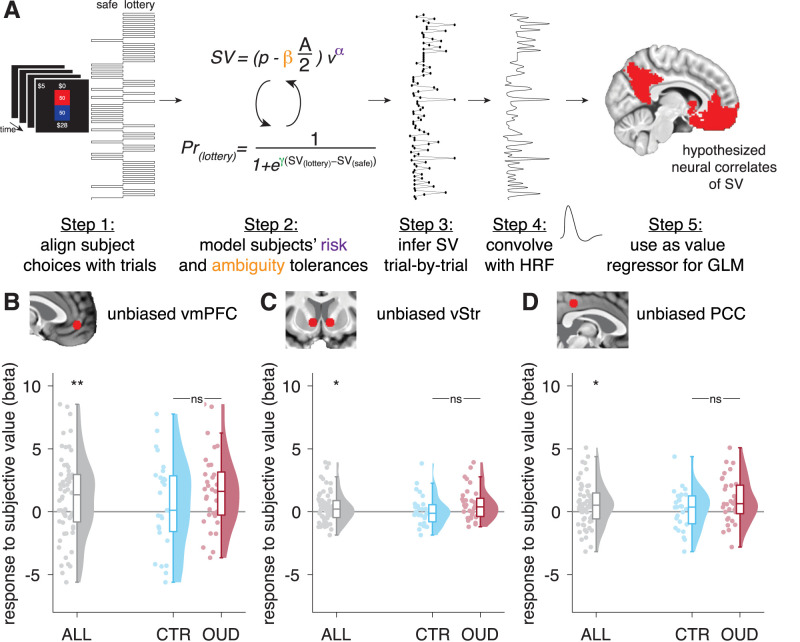
Neural correlates of SV under uncertainty in canonical value regions (univariate results). ***A***, Model-based fMRI methods. Steps 1, 2 involved estimating individual risk and ambiguity tolerance parameters from choice behavior on the task. Using group medians for these parameters and our utility model (Eq. 1), Step 3 generated an inferred SV of the lottery (or chosen) option trial-by-trial for each participant. This variable was convolved with the hemodynamic response function (HRF) to act as a parametric modulator/regressor of neural activity at decision period in a GLM (Step 4), which is hypothesized to reveal univariate correlates of SV in canonical value regions (Step 5). ***B–D***, Mean responses to SV (betas from our primary model, GLM_SVlot_, using group median parameters) across all participants (gray, two-tailed one-sample *t* test) and in comparison controls (blue) and participants with OUD (red; two-tailed two–sample *t* test) within unbiased 5 mm radius spheres in the (***B***) vmPFC, (***C***) bilateral ventral striatum (vStr), and (***D***) PCC. Dots are individual participant data, and sky plots represent group density functions. Boxplots represent median and interquartile range. See also Figure S1 and Table S1. **p < 0.01; *p < 0.05; ns, not significant.

GLM_SVlot_ (context-independent encoding of SV) included condition (indicator) regressors for decision and missing trials (no response within 2 s) and a single parametric modulator for decision based on the SV of the lottery option on each trial. SV was inferred from the computational model (Eq. 1; Fig. 2*A*) using the median known-risk tolerance (*α*) and ambiguity tolerance (*β*) parameters across participants, as these did not differ between groups (see Results) and as is a common practice in model-based fMRI studies to minimize noise due to uncertainty around individual parameter estimates, particularly in modest-sized samples (Wilson and Niv, 2015; Lebreton et al., 2019). The regressor was normalized within each run by scaling values to [0,1] based on the maximum SV encountered in that run. We also considered alternative GLMs in which we modeled the SV of the chosen option on each trial rather than the lottery option (i.e., if the safe option was chosen, we used SV_(safe)_, and if the lottery option was chosen, we used SV_(lottery)_), as well as choice difficulty-related responses (i.e., the absolute difference between SV_(safe)_ and SV_(lottery)_), and all models using individual participant-specific parameters (Text S1). These models all yielded similar results (Fig. S1, Table S1), and therefore for ease of interpretation and comparison to multivariate analyses, we focus here on GLM_SVlot_ using group median parameters.

Second-level (group) analyses tested for main task effects as well as for differences between OUD participants and controls and between uncertainty-averse and uncertainty-seeking participants, using one-sample *t* tests (task effect) and two-sample *t* tests (between-person differences) on averaged beta estimates from GLM_SVlot_. For this purpose, we created unbiased spheres with a 5 mm radius centered around coordinates from a published meta-analysis of the brain's canonical valuation system [Bartra et al., 2013; MNI coordinates, vmPFC (*x* = −1; *y* = 46; *z* = −7), bilateral ventral striatum (*x* = ±10; *y* = 10; *z* = −4), and PCC (*x* = −4; *y* = −30; *z* = 36)]. Results were considered significant at *p* < 0.05. For exploratory whole-brain analyses, results were thresholded at voxel-level *p* < 0.001 uncorrected, and significant clusters were defined as surviving *p* < 0.05 family-wise error correction at the cluster-level.

GLM_beta-series_ (trial-specific activity, “beta-series” GLM) used separate condition regressors for each trial. No parametric modulators were included. This model was used in the multivariate analyses, described below, and estimated on unsmoothed data to maintain existing voxel patterns.

#### Multivariate BOLD fMRI analyses

**Figure 3.**
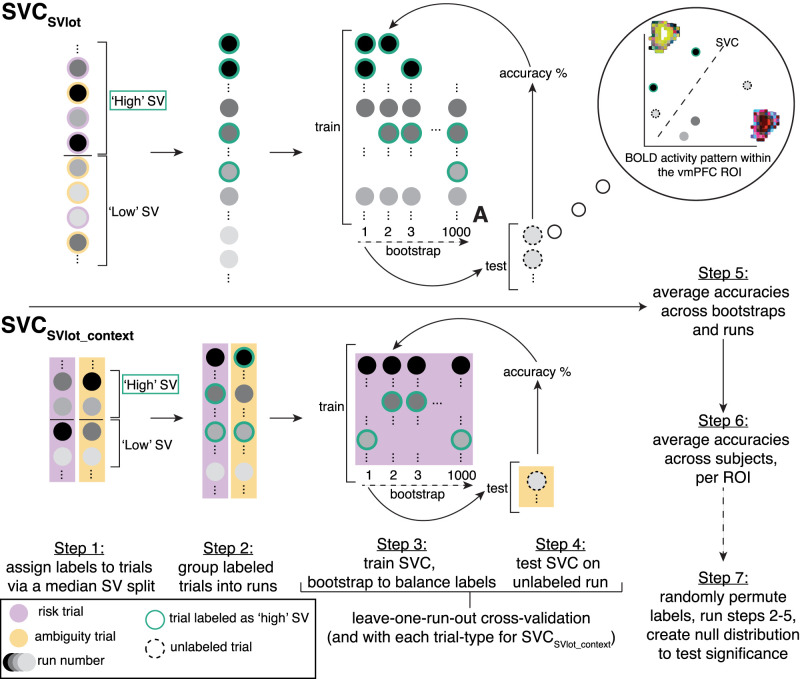
MVPA classification methods. For SVC_SVlot_ (top), Step 1 involved labeling trials as either “high” or “low” SV, based on our utility model (Eq. 1) and participant-specific parameters for risk and ambiguity tolerance. Step 2 grouped trials by run (four runs) in preparation for leave-one-run-out (4-fold) cross-validation involving training a SVC on 3 of the 4 runs (Step 3) and testing it on unlabeled data in the held-out run (Step 4). This procedure was repeated 1,000 times (bootstrapping) after first balancing the labeled data in the training set. Accuracies were averaged across bootstraps and folds per participant (Step 5) and then across participants (Step 6) for each ROI. Statistical significance (Step 7) was assessed by randomly permuting the SV labels (1,000 times) before repeating Steps 2–5 to create an empirical null distribution. For SVC_SVlot_context_ (bottom), the same general procedures followed except that trials were first separated by uncertainty context (known-risk, in purple, and ambiguity, in orange) and, for Steps 3, 4, the training set only contained data from one context (e.g., risk) while the testing set (held-out run) only contained data from the other context (e.g., ambiguity).

Given cross-species evidence of nonuniform and broadly distributed value encoding (Vickery et al., 2011; Hunt and Hayden, 2017; Kahnt, 2018; Overton et al., 2025), we used multivoxel pattern analysis (MVPA) as a complementary and potentially more sensitive approach to univariate analyses (Haxby et al., 2001), to examine brain activity patterns reliably associated with SV (Fig. 3). MVPA increases sensitivity by leveraging distributed voxel patterns rather than individual voxel activations and increases specificity by detecting fine-grained informational content rather than activation magnitude alone. Also, unlike univariate methods, MVPA emphasizes discriminability over directionality and relies on any differences, including those related to signal and noise, for purposes of discriminability and prediction of a stimulus/condition of interest (Hebart and Baker, 2018). While voxels identified as relevant in univariate analyses are often also informative for MVPA, the reverse is not necessarily true as MVPA can detect subtle, distributed signals that univariate approaches miss (Jimura and Poldrack, 2012).

**Figure 4.**
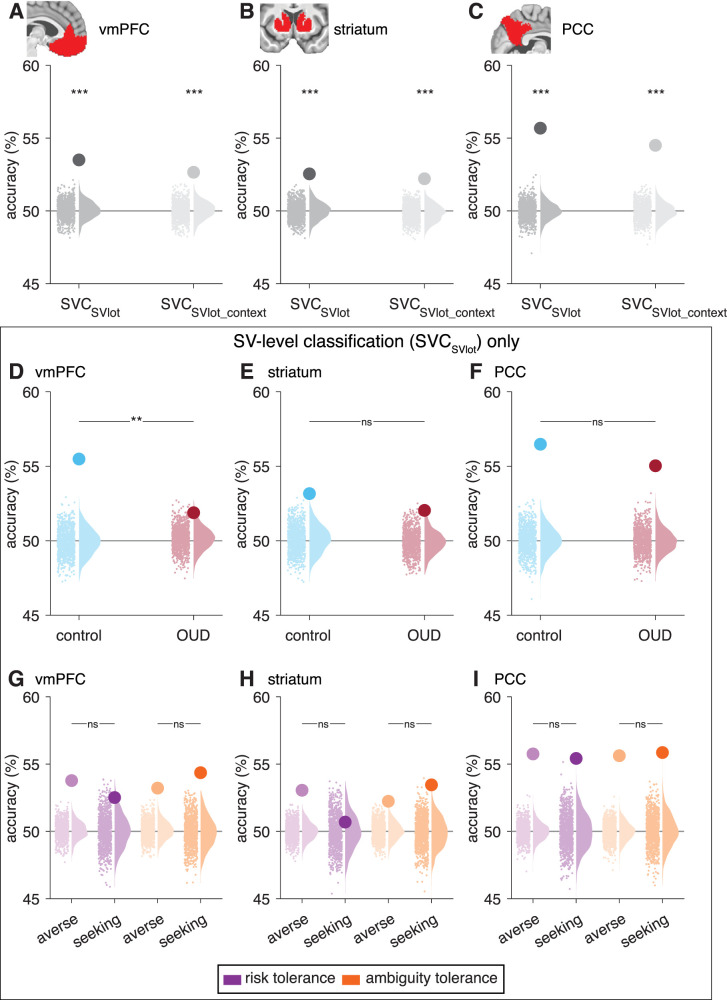
Neural correlates of SV under uncertainty in canonical value regions (multivariate results). ***A–C***, Mean classification accuracy across all participants (large dots) for decoding between high and low SV trials (SVC_SVlot_; dark gray) and between known-risk and ambiguity trials for high versus low SV (SVC_SVlot_context_; light gray) within atlas-based ROI masks in (***A***) vmPFC, (***B***) striatum, and (***C***) PCC. Mean classification accuracy for decoding between high and low SV trials (SVC_SVlot_) in (***D–F***) comparison controls (blue) and participants with OUD (red) and (***G–I***) risk-averse (light purple) or risk-seeking (dark purple) and ambiguity-averse (light orange) or ambiguity-seeking (dark orange) participants, within the vmPFC, striatum, and PCC. Gray horizontal lines represent theoretical chance level accuracy (50%). Small dots are individual participant permuted data, and sky plots represent group density functions for the null distribution. ***p < 0.001; **p < 0.01; ns, not significant.

To this end, we first combined masks from the previously validated Schaefer-200 cortical (Schaefer et al., 2018) and Tian-16 subcortical (Tian et al., 2020) atlases to create regions of interest (ROIs) for the vmPFC, striatum, and PCC that overlapped with meta-analytic findings mapping the brain's valuation system (Bartra et al., 2013; Fig. 4*A*–*C*). Both atlases are derived from functional connectivity-based parcellations of the human brain, with Tian et al. (2020) also considering broad-scale anatomical boundaries. Second, consistent with a role in numerous cognitive and sensory functions, previous research has shown that reward signals can be decoded from over 80% of the human brain in healthy young adults (Vickery et al., 2011). Therefore, to further investigate distributed activity patterns associated with value across the entire brain (beyond canonical value regions), we used a parcel-wise MVPA approach that included as ROIs all 200 cortical parcel regions, grouped into seven large-scale networks for interpretation (Yeo et al., 2011), and all 16 subcortical parcel regions as their own network. MVPA was carried out using *The Decoding Toolbox* (Hebart et al., 2015) after first transforming all ROIs to the MNI152NLin2009cAsym space.

For each participant, a binary class support vector classifier (SVC) with *C* = 1 was fit separately for each ROI using the trial-specific parameter estimates (from GLM_beta-series_) per voxel within that ROI as features. We used SVC rather than support vector regression to maintain computational simplicity, similar to previous decision neuroscience studies (Kahnt et al., 2010; McNamee et al., 2013; Suzuki et al., 2017; Xue et al., 2022). Missing response trials were excluded from analysis. Additionally, to account for the increased noise of single-trial analyses, trials where the mean beta for each ROI was outside three standard deviations from the ROI's mean across trials were removed from analysis (an average of 0.628 trials per participant per ROI; range, 0–4), with no difference in this number between groups (*t*_(58)_ > 0.184; *p* > 0.171). We estimated two primary models.

SVC_SVlot_ (SV level classification) aimed at distinguishing activity patterns associated with “low” and “high” SV. We labeled each trial using a median split on each participant's normalized SVs of the lottery option, based on their individual known-risk (*α*) and ambiguity (*β*) tolerance parameters. Individual-specific parameters were used instead of group medians (as in univariate analyses) to allow for single-participant-level decoding that aligns with individual preferences. Trials below a participant's overall median lottery SV across runs were labeled as low, while those above were labeled as high. Trials with SVs equal to the median were assigned to whichever category helped balance the labels. Classifier performance was evaluated using a leave-one-run-out (fourfold) cross-validation procedure. Because labels were slightly imbalanced for most participants, we used a bootstrapping procedure (with 1,000 repetitions) to equalize label counts by randomly removing samples from the training set (Fig. 3), following prior studies (Suzuki et al., 2017). Cross-validation with bootstrapping was implemented using the *make_design_boot_cv* function in *The Decoding Toolbox*, and the SVC was performed on the balanced data.

SVC_SVlot_context_ (SV level cross-classification) aimed to identify activity patterns distinguishing “low” and “high” SV across uncertainty contexts (known-risk vs ambiguity). Here, the SVC was trained on low versus high SV trials from one context (e.g., ambiguity) in three runs and tested on trials from the other context (e.g., known-risk) in the held-out run, maintaining consistency with SVC_SVlot_. SV labels were assigned using a median split on normalized data separately within each trial type/context. As before, to account for slight label imbalances, we used a bootstrapping procedure (1,000 repetitions; Fig. 3). Cross-classification decoding with bootstrapping was implemented by modifying the *make_design_xclass_cv* function in *The Decoding Toolbox*.

For each ROI, accuracies were averaged across folds per participant and then across participants to obtain a group-level cross–validated classifier accuracy. Significance was assessed using nonparametric permutation testing. For each participant, we randomly permuted the training labels (low vs high SV), tested model performance on the left-out data, and then averaged model accuracies across validation folds. This procedure was repeated 1,000 times to obtain an empirical null distribution of classification accuracies per participant, which was then averaged across participants. Permuted *p* values were calculated as the proportion of null-distributed classification accuracies that were greater than the observed accuracy, with values <0.05 considered significant.

To compare decoding accuracies between groups (OUD vs control participants, uncertainty-seeking vs uncertainty-averse participants) within canonical value ROIs, we similarly employed permutation testing. Null distributions of group differences were obtained by randomly permuting the group labels of participants’ cross-validation scores 1,000 times, and permuted *p* values were calculated as the proportion of null-distributed differences that were greater than the observed difference, with values <0.05 considered significant.

To assess the “widespreadness” of value signals beyond canonical value regions and compare groups, we extended this approach as follows. First, we computed permuted *p* values for each ROI (216 total) and group (OUD, control). Second, we calculated the proportion of significant ROIs within each large-scale network for each group. As we did not have a priori hypotheses for this analysis, we report results using three different significance thresholds: *p* < 0.05, *p* < 0.01, and *p* < 0.001. Lastly, we repeated these steps with permuted group labels (1,000 times) to generate a null distribution of group differences in our “widespreadness” index (proportion of significant ROIs). As above, permuted *p* values were calculated as the proportion of null-distributed differences at each threshold that were greater than the observed difference, with values <0.05 considered significant.

## Results

### Choice behavior is sensitive to explicit task features and reflects individual uncertainty preferences

As expected, participants were more likely to choose the lottery options when they offered higher potential reward (risk trials, *B* = 2.175; 95% CI [1.893, 2.456]; *t*_(3,725)_ = 15.16; *p* = 2.160 × 10^−50^; ambiguity trials, *B* = 1.781; 95% CI [1.564, 1.998]; *t*_(3,494)_ = 16.10; *p* = 2.607 × 10^−56^) and higher probability of reward (25, 50, or 75%; *B* = 1.703; 95% CI [1.497, 1.909]; *t*_(3,725)_ = 16.20; *p* = 4.340 × 10^−57^) and were associated with lower levels of ambiguity (74, 50, or 24% occlusion; *B* = −0.664; 95% CI [−0.821, −0.508]; *t*_(3,494)_ = −8.333; *p* = 1.119 × 10^−16^; Fig. 1*C*,*D*). These effects were qualified by significant interactions between reward value and lottery probability/ambiguity level (v*p, *B* = 0.982; 95% CI [0.741, 1.222]; *t*_(3,725)_ = 8.007; *p* = 1.560 × 10^−15^; v*A, *B* = −0.306; 95% CI [−0.486, −0.127]; *t*_(3,494)_ = −3.344; *p* = 0.0008), as well as a three-way interaction with diagnosis for risk trials (v*p*DX, *B* = −0.618; 95% CI [−0.903, −0.332]; *t*_(3,725)_ = −4.245; *p* = 2.237 × 10^−5^; v*A*DX, *p* = 0.289). Additionally, we compared the selection of first-order stochastically dominated lotteries (where *v* was less than the safe $5 option) and found that the number of such “error” trials did not differ between groups (mean [SE] in controls, 1.185 [0.450]; OUD, 1.758 [0.399]; *t*_(58)_ = −0.954; *p* = 0.344). Thus, although both groups considered the lottery characteristics in their decision process (as evidenced by the main and interactive effects on choice within each group; Fig. 1*E*), controls were more sensitive to risk–reward trade-offs, suggesting subtle group differences in the decision process for risky, though not ambiguous, lotteries.

To quantify individual differences in uncertainty preferences, we fit a modified expected utility model to each participant's trial-by-trial choices to estimate known-risk (*α*) and ambiguity (*β*) tolerance. Model fits were moderate to good (controls, mean [SE] pseudo-*R*^2^ = 0.582 [0.040]; OUD, mean pseudo-*R*^2^ = 0.518 [0.044]) and did not differ significantly between groups (*t*_(58)_ = −0.938; *p* = 0.352). Individual proportion of lottery choices correlated strongly with the corresponding model-based parameters, for both known-risk (controls, *R_s_* = 0.968; *p* = 1.634 × 10^−16^; OUD, *R_s_* = 0.712; *p* = 3.325 × 10^−6^) and ambiguity (controls, *R_s_* = 0.885; *p* = 1.268 × 10^−6^; OUD, *R_s_* = 0.653; *p* = 5.710 × 10^−5^), together supporting that our model adequately captured behavioral choice patterns and preferences in both groups.

Despite individual variability, participants were generally risk-averse (*α *< 1; controls, median [IQR] = 0.599 [0.525]; OUD, median = 0.628 [0.395]) and ambiguity-averse (−*β *< 0; controls, median = −0.524 [0.988]; OUD, median = −0.352 [1.424]), with no significant group differences in either parameter (*α*, *z*_(58)_ = −0.639; *p* = 0.523; −*β*, *z*_(58)_ = −0.253; *p* = 0.801; Fig. 1*F*,*G*). There were also no significant group differences in the noise parameter (*γ*, *z*_(58)_ = 1.070; *p* = 0.285). Consistent with prior studies (Levy et al., 2010; Tymula et al., 2012; Pushkarskaya et al., 2015; Ruderman et al., 2016; Konova et al., 2020; Raio et al., 2022), *α* and −*β* were only weakly correlated (controls, *R_s_* = 0.201; *p* = 0.312; OUD, *R_s_* = 0.237; *p* = 0.184; Fig. 1*H*), indicating they captured largely distinct components of an individual's global risk-taking propensity.

### Univariate correlates of SV in canonical value regions largely do not differ between groups

Focusing on the three canonical value areas—vmPFC, ventral striatum, and PCC—we used unbiased analyses to test for scaling of their activity by SV of the lottery option (regardless of the type of uncertainty; GLM_SVlot_) and assess for potential individual differences in this response. As expected, we found SV effects in all three regions (one-sample *t* test on average betas; *t*_(59)_ > 2.30; *p* < 0.025), but there were no significant differences between groups (two-sample *t* test, vmPFC, *t*_(58)_ = −1.155; *p* = 0.253; bilateral striatum, *t*_(58)_ = −1.540; *p* = 0.129; PCC, *t*_(58)_ = −1.661; *p* = 0.102; Fig. 2*B*–*D*). To further investigate the impact of individual uncertainty preferences, we compared instead uncertainty-averse versus uncertainty-seeking participants, regardless of diagnosis, and found no significant differences (for risk-averse vs risk-seeking groups, *p* > 0.110; for ambiguity-averse vs ambiguity-seeking groups, *p* > 0.149). We also did not find evidence that clinical characteristics within the OUD group, including concomitant opioid use or the duration of current treatment, significantly modulated these responses (Text S1).

Whole-brain analyses across all participants confirmed that trial-wise SV correlated positively and selectively with BOLD signal in the PCC ([−1, −51, 20]; *t* = 4.02; *k* = 84; cluster-level *p*_FWEcorr_ = 0.044), while effects in the vmPFC and striatum did not reach cluster-level corrected significance. Additionally, no significant differences in canonical value regions were observed in the whole-brain results between OUD and comparison control participants, for our primary model or any of the other univariate GLMs considered (Table S1).

Together, these results suggest that, when assessed via univariate analyses, OUD participants engage SV-based processes during risky decision-making, similar to comparison controls, and that individual preferences for risk/ambiguity seemingly also do not impact this neural computation.

### Multivariate representations of SV are less reliably decodable in the vmPFC in OUD

As a potentially more sensitive approach, and recognizing that value signals manifest in nonuniform regional responses (Kahnt, 2018), we used MVPA to identify brain activity patterns distinguishing high versus low SV. This was done via a median split of trials, labeling them as either high SV or low SV, then using a leave-one-run-out (four-fold) cross-validation with 1,000 bootstraps (Fig. 3). Permutation testing was then used to assess significance. Across all participants, the SV level could be significantly decoded from activity in the vmPFC, striatum, and PCC (the SVC_SVlot_ model within atlas-defined ROIs, all *p*_perm_test_ < 0.0001; Fig. 4*A*–*C*). Cross-classification analyses further showed that SV patterns generalized across uncertainty contexts (known-risk vs ambiguity), with successful decoding in all three canonical value regions when training on one context and testing on the other (SVC_SVlot_context_; all *p*_perm_test_ < 0.0001; Fig. 4*A*–*C*). These results extend prior cross-species work showing that decision variables can be reliably decoded from patterns of activity in canonical value regions and support the idea of a context-general representation of SV in the context of monetary rewards (McNamee et al., 2013), consistent with theories of a unified value signal.

Testing for individual differences, we found the SV level (from SVC_SVlot_) could be significantly decoded from all three canonical value regions in both controls (*p*_perm_test_ < 0.0001) and OUD participants (*p*_perm_test_ < 0.020; Fig. 4*D*–*F*). However, decoding accuracies in the vmPFC (*p*_perm_test_group_ = 0.005), although not the striatum (*p*_perm_test_group_ = 0.172) or PCC (*p*_perm_test_group_ = 0.173), were significantly lower in OUD participants. There were no significant differences between risk-averse and risk-seeking participants (*p*_perm_test_group_ > 0.573) or ambiguity-averse and ambiguity-seeking participants (*p*_perm_test_group_ > 0.185; Fig. 4*G*–*I*). Thus, while the core neural representation of SV appears preserved across groups, OUD participants show attenuated vmPFC decoding, suggesting a less reliable representation of SV information.

### Brain-wide decoding reveals less distributed representations of SV in OUD

**Figure 5.**
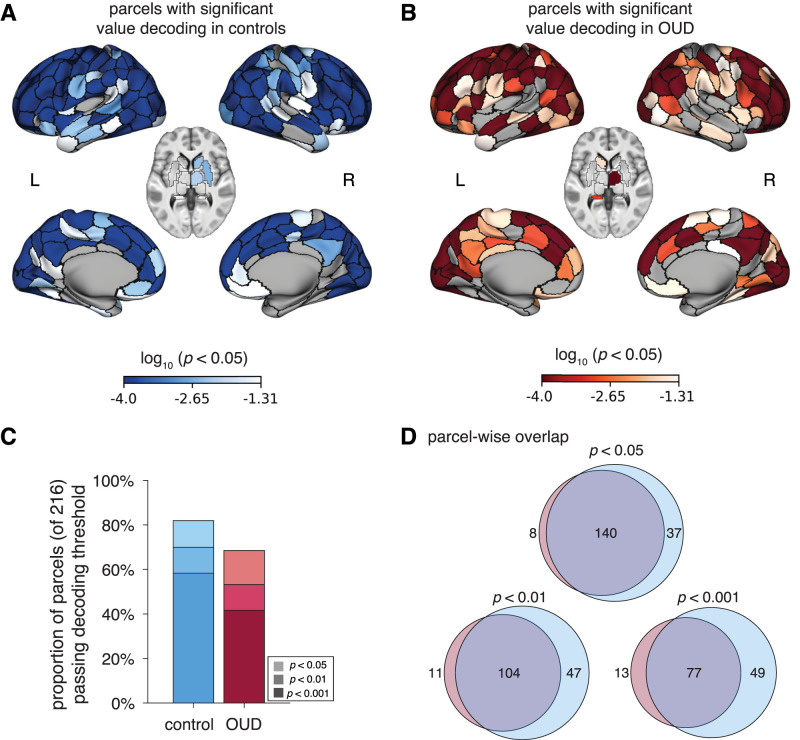
Neural correlates of SV under uncertainty across the brain (multivariate results). ***A***, ***B***, Regions across 200 cortical parcels (Schaefer et al., 2018) and 16 subcortical parcels (Tian et al., 2020) with significant SV decoding shaded by each region's log_10_-transformed permuted *p*_perm_test_ values in (***A***) comparison controls (blue) and (***B***) participants with OUD (red). Nonsignificant regions are left gray. ***C***, Proportion of all 216 parcels passing each significance threshold (*p*_perm_test_ < 0.05; *p*_perm_test_ < 0.01; *p*_perm_test_ < 0.001) in comparison controls (blue) and participants with OUD (red). ***D***, The overlap of significant parcels between groups for each significance threshold.

**Figure 6.**
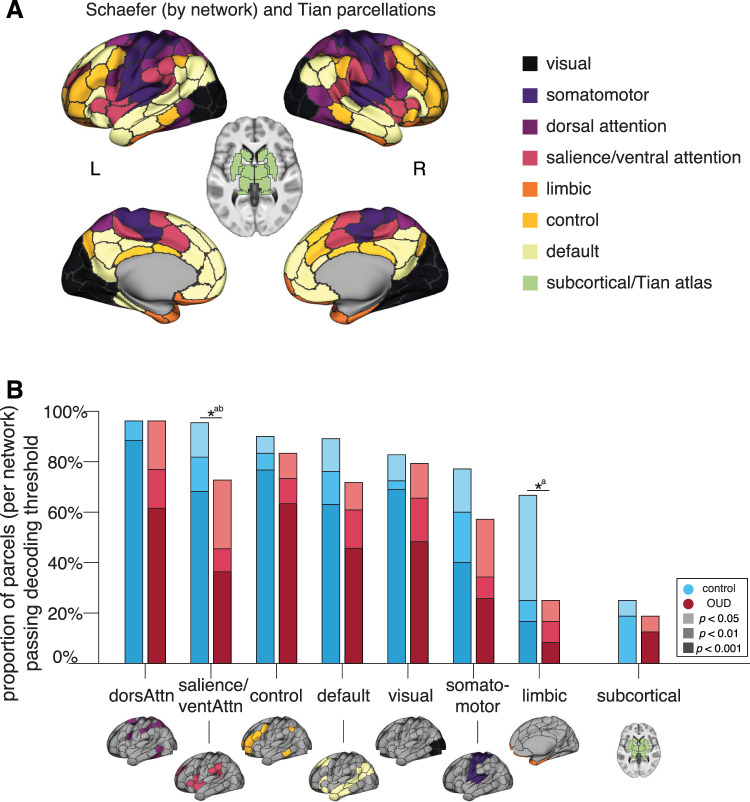
Network analysis of parcels with significant SV decoding. ***A***, Regions across 200 cortical parcels (Schaefer et al., 2018) grouped into seven large-scale brain networks following Yeo et al. (2011). Subcortical parcels (Tian et al., 2020) are shown as their own (8th) network. ***B***, Proportion of all parcels within each network with significant value decoding passing each significance threshold (*p*_perm_test_ < 0.05; *p*_perm_test_ < 0.01; *p*_perm_test_ < 0.001) in comparison controls (blue) and participants with OUD (red). *p_perm_test_group_ < 0.05 at different decoding thresholds (a, *p*_perm_test_ < 0.05; b, *p*_perm_test_ < 0.01).

To assess the extent to which value information is distributed throughout the brain, building on prior findings (Vickery et al., 2011; Hunt and Hayden, 2017; Kahnt, 2018; Overton et al., 2025), we ran the decoding analysis within 216 cortical and subcortical parcel ROIs (Fig. 5*A*,*B*). Across all participants, SV could be significantly decoded from over 86.6% of all ROIs at the *p*_perm_test_ < 0.05 threshold, 76.9% at the *p*_perm_test_ < 0.01 threshold, and 66.2% at the *p*_perm_test_ < 0.001 threshold. In controls, these numbers ranged from 81.9% (at *p*_perm_test_ < 0.05) to 58.3% (at *p*_perm_test_ < 0.001) and in OUD participants from 68.5% (at *p*_perm_test_ < 0.05) to 41.7% (at *p*_perm_test_ < 0.001; Fig. 5*C*). There was moderate-to-high overlap (from 75.7 to 55.4% depending on the threshold applied) in which parcels had significant value decoding in both groups (Fig. 5*D*). Significant regions belonged to each of the large-scale networks, with not any one network demonstrating preferential involvement (Fig. 6*A*). Although decoding accuracies can be influenced by the ROI size (Haynes, 2015), the average parcel size within each network was comparable, with the exception of the subcortical network, which had, on average, smaller regions than the other networks. Therefore, differences between the subcortical and other networks should be cautiously interpreted.

To assess for group differences in the “widespreadness” of value representations, we compared the proportion of parcel ROIs with significant value decoding within each large-scale network (Fig. 6*B*). Relative to controls, participants with OUD demonstrated a smaller proportion of regions with significant value decoding within the salience/ventral-attention network, at both the *p*_perm_test_ < 0.05 (Δ = 22.7%; *p*_perm_test_group_ = 0.047) and *p*_perm_test_ < 0.01 thresholds (Δ = 36.4%; *p*_perm_test_group_ = 0.021; at *p*_perm_test_ < 0.001, the difference was trending, *p*_perm_test_group_ = 0.084), and within the limbic network, at the *p*_perm_test_ < 0.05 threshold (Δ= 41.7%; *p*_perm_test_group_ = 0.033; differences were nonsignificant at the other two thresholds, *p*_perm_test_group_ > 0.178). No other networks/thresholds reached significance.

## Discussion

We used both univariate and multivariate fMRI methods and an economic model of SV or “utility” to evaluate whether people with OUD demonstrate an overt disconnect between their decisions and the neural computation and encoding of a SV signal that should guide choice behavior. We did not find strong support for such an impairment. Instead, we found more nuanced differences in how reliably decodable value signals are within the vmPFC and across several large-scale brain networks. These findings suggest that people with OUD engage a neurocomputationally similar process during decision-making to controls—integrating information about reward, uncertainty, and their idiosyncratic preferences—but may demonstrate a less robust, and spatially more restricted, representation of value.

Consistent with an extensive literature in healthy individuals (Levy and Glimcher, 2012; Bartra et al., 2013; Clithero and Rangel, 2014; Lopez-Persem et al., 2020), we found that SV was represented in the vmPFC, striatum, and PCC, regions forming the core of the brain's canonical valuation system, in both univariate and multivariate decoding analyses and across both groups. Follow-up analyses revealed this value signal was also preserved across individuals with varying risk-taking propensities and was domain-general, common to two types of uncertainty (known-risk and ambiguity). Nevertheless, we observed comparatively decreased decoding accuracies in OUD participants in the vmPFC, suggesting patterns distinguishing between high and low SV in this region are less reliably separable. Since decoding accuracy tends to be higher for attended than unattended (i.e., behaviorally irrelevant) information (Christophel et al., 2018), one possibility is that OUD participants paid less attention to the lottery characteristics. This aligns with their somewhat weaker, although still significant, behavioral sensitivity to the explicitly stated rewards and probabilities in the task. Alternatively, reduced decoding accuracy in the vmPFC may reflect noisier or less stable population-level value representations, potentially due to greater variability in signal, shifting single-neuron-level contributions to the value representation, or changes in the noise structure over time. Since univariate and multivariate approaches capture different aspects of neural coding (Hebart and Baker, 2018), such differences underscore the added sensitivity of multivariate methods to subtle disruptions in representational fidelity.

Beyond canonical value regions, our brain-wide decoding analyses revealed that SV information is distributed across nearly the entire brain, spanning all major large-scale networks without preferential involvement of any single network. Depending on the significance threshold applied, value could be decoded from up to 66–87% of all brain parcels examined. This finding extends prior work across species and measurement techniques (Vickery et al., 2011; Hunt and Hayden, 2017; Overton et al., 2025), which suggests decision variables, including value, are represented more broadly than traditionally assumed, even in sensory and motor cortices. The precise functional role of this widespread representation remains unclear, but one possibility is that distributed encoding enhances robustness, helping preserve the fidelity of important signals like value signals. This redundancy may, in turn, facilitate adaptive decision-making behavior and support integration with other cognitive functions.

Notably, value signals were consistently less widespread in OUD participants and 23–42% less decodable from limbic and salience/ventral-attention network regions. The limbic network comprises regions centrally implicated in emotion and memory (Papez, 1937; Maclean, 1949; Catani et al., 2013) and has a long-recognized role in the maintenance of substance use disorders (Koob and Volkow, 2010; Volkow et al., 2019; Pando-Naude et al., 2021). Furthermore, the salience/ventral-attention network integrates relevant stimuli with internal goals (Seeley, 2019) and has emerged as a common neural substrate of nearly all mental health disorders (Goodkind et al., 2015). Reduced decoding accuracies in patients could reflect functional alterations within these networks, including in their interactions and communication with value regions (Haber and Knutson, 2010; Zhang et al., 2017), and future studies using methods such as activity flow mapping (Cocuzza et al., 2022) may be of particular interest in addressing this possibility. Such a global shift could also be explained by reduced neuromodulator action in participants with OUD that fails to tune the precision of neural representations (Servan-Schreiber et al., 1990; Aston-Jones and Cohen, 2005), particularly of dopamine, which has been shown to enhance decoding of reward information in the cortex (Kahnt et al., 2015). Alternatively, it could reflect morphological neuroadaptations and reorganization of parcel and/or network boundaries, as supported by recent findings in other psychopathologies (Lynch et al., 2024). Regardless of the underlying mechanism, these data highlight differences in the fidelity and network-level distribution of value signals in OUD, with potential implications for how these signals might influence broader cognition and behavior in this population.

Given the widespread effects observed, a potential concern is that our analyses are picking up on features other than value, like stimulus salience. This is unlikely given that the lottery images within an uncertainty context were visually very similar and that value signals were cross-decoded between contexts despite differences in visual characteristics (the presence of an occluder in the ambiguity trials). Moreover, our analyses focused on high versus low SV, all in the gain domain and during choice deliberation, providing a stringent test of these effects and avoiding some of the issues that can confound comparisons between, e.g., reward and nonreward or when the outcomes of choices are revealed (Vickery et al., 2011). Nevertheless, high-value options naturally command more attention, and it may not be possible to completely separate out attention from value in the current design.

A few other limitations should be acknowledged. Our modest sample size and limited female participation require that future studies replicate our findings in larger, more sex-/gender-balanced samples. In this vein, we did not observe significant group differences in uncertainty preferences even though, historically, people with OUD have demonstrated an increased tolerance for risk on similar tasks (Gowin et al., 2013; Biernacki et al., 2016; Chen et al., 2020; Konova et al., 2020). Such differences tend to be moderate to small in size and may be influenced by sample composition, requiring larger or longitudinal datasets to detect reliably. Furthermore, most prior studies focused on participants receiving methadone (Biernacki et al., 2016; Konova et al., 2020), whereas our sample was primarily treated with buprenorphine. Given some documented differences between these medications in clinical outcome (Degenhardt et al., 2023) and impact on decision-making behavior (Pirastu et al., 2006), it is also possible that treatment-related factors could have contributed to the absence of behavioral differences in our study. Nevertheless, we observed a wide range of behavioral preferences in both study groups, which allowed us to explore their impact on our results. We did not find evidence that individual known-risk or ambiguity tolerance levels led to differential neural correlates, indicating results generalize even when preferences differ, in line with prior studies (Kable and Glimcher, 2007; Levy et al., 2010; Konova et al., 2023). Furthermore, even in the absence of behavioral differences, we observed differences in multivariate decoding in patients. It remains possible that the treatment status of our OUD sample could have masked, or even helped repair, an underlying impairment. Although we did not find evidence consistent with this possibility, fully addressing this question would require comparison to nontreatment-engaged individuals or a longitudinal (pre/post) treatment design. We note, however, despite being treatment-engaged, clinically, our sample presented as quite severe with chronic opioid use (including the intravenous use and history of overdose) and moderate levels of opioid-related symptomatology and concomitant use (Table 1). That only relatively subtle neural alterations emerged in this context is, in itself, striking.

In summary, our findings provide novel evidence that neural subjective valuation processes are largely intact in OUD but are less reliable and less broadly distributed across the brain than in controls. This suggests that value-based decision-making mechanisms, as understood in healthy individuals, still apply to individuals with OUD but may be functionally altered in ways that introduce noise and reduce reliability. At a theoretical level, these results reinforce the translational relevance of standard neurocomputational decision-making models for understanding addiction and suggest new directions for intervention. Specifically, the reduced reliability of neural value representations could be a meaningful target for treatment. Additionally, since these differences were only detectable using MVPA and not univariate measures or behavior alone, such multivariate patterns may provide a valuable avenue for biomarker development. More broadly, our findings emphasize the distributed nature of decision-related neural processing. The widespread distribution of value signals throughout the brain underscores how decision-making is not confined to a single region, cell type, or computation. Instead, this distributed architecture likely serves an adaptive function, integrating value with other cognitive and sensory processes to support flexible behavior. Together, our findings offer a more nuanced framework for both research and treatment in addiction. Moving beyond neuropsychological models of overt impairment and traditional decision-making centers, they shift focus instead on disruptions in the computational fidelity of value representations and their distribution across a broader range of neural and cognitive systems.
